# Selective Retinoic Acid Receptor γ Antagonist 7C is a Potent Enhancer of BMP-Induced Ectopic Endochondral Bone Formation

**DOI:** 10.3389/fcell.2022.802699

**Published:** 2022-03-14

**Authors:** Daisuke Tateiwa, Takashi Kaito, Kunihiko Hashimoto, Rintaro Okada, Joe Kodama, Junichi Kushioka, Zeynep Bal, Hiroyuki Tsukazaki, Shinichi Nakagawa, Yuichiro Ukon, Hiromasa Hirai, Hongying Tian, Ivan Alferiev, Michael Chorny, Satoru Otsuru, Seiji Okada, Masahiro Iwamoto

**Affiliations:** ^1^ Department of Orthopaedic Surgery, Osaka University Graduate School of Medicine, Suita, Japan; ^2^ Department of Orthopaedic Surgery, Osaka Second Police Hospital, Osaka, Japan; ^3^ Department of Orthopaedic Surgery, Mino Municipal Hospital, Mino, Japan; ^4^ Department of Orthopaedics, University of Maryland School of Medicine, Baltimore, MD, United States; ^5^ Department of Orthopaedic Surgery, Kansai Rosai Hospital, Amagasaki, Japan; ^6^ Department of Pediatrics, The Children’s Hospital of Philadelphia, Philadelphia, PA, United States

**Keywords:** bone morphogenetic protein, retinoic acid receptor γ, endochondral bone formation, bone regeneration, Bmp/Smad signaling, RARγ inverse agonist

## Abstract

Bone morphogenetic proteins (BMPs) have been clinically applied for induction of bone formation in musculoskeletal disorders such as critical-sized bone defects, nonunions, and spinal fusion surgeries. However, the use of supraphysiological doses of BMP caused adverse events, which were sometimes life-threatening. Therefore, safer treatment strategies for bone regeneration have been sought for decades. Systemic administration of a potent selective antagonist of retinoic acid nuclear receptor gamma (RARγ) (7C) stimulated BMP-induced ectopic bone formation. In this study, we developed 7C-loaded poly lactic nanoparticles (7C-NPs) and examined whether local application of 7C enhances BMP-induced bone regeneration. The collagen sponge discs that absorbed recombinant human (rh) BMP-2 were implanted into the dorsal fascia of young adult mice to induce ectopic bone. The combination of rhBMP-2 and 7C-NP markedly increased the total bone volume and thickness of the bone shell of the ectopic bone in a dose-dependent manner compared to those with rhBMP-2 only. 7C stimulated sulfated proteoglycan production, expression of chondrogenic marker genes, and Sox9 reporter activity in both chondrogenic cells and MSCs. The findings suggest that selective RARγ antagonist 7C or the related compounds potentiate the bone inductive ability of rhBMP-2, as well as support any future research to improve the BMP-2 based bone regeneration procedures in a safe and efficient manner.

## 1 Introduction

Treatment for large bone defects caused by tumor resection or complex fracture remains a challenge in the field of orthopedics. Autogenous bone grafting has been the gold standard, but the application is hampered by limited availability and donor site morbidity (Laurie et al., 1984; Betz, 2002). Bone morphogenetic proteins (BMPs) which belong to the transforming growth factor superfamily have been attracting attention as a novel osteoinductive grafting material (Urist, 1965; Wozney et al., 1988; Burkus et al., 2002; Lo et al., 2012).

However, it is challenging to strike a balance between ensuring sufficient BMP bone-inducing capacity and suppressing the side effects of BMP. The use of BMP with supra-physiological high dose is reported to correlate with dose-dependent side effects such as inflammation, soft tissue edema, unintended ectopic bone formation, and the deteriorated quality of newly formed bone (Cahill et al., 2009; Zara et al., 2011; James et al., 2016). On the other hand, in a study of human open tibial fractures, the rate of non-union was increased by more than 40% after low-dose BMP treatment (Govender et al., 2002). There is a strong need for a method that efficiently induces bone regeneration with low doses of BMPs.

Retinoic acid is an active metabolite of vitamin A, and it plays a critical role in cellular differentiation, embryogenesis, and maintaining homeostasis (Chambon, 1996; Henning et al., 2015). Among three types of retinoic acid receptors (RARα, RARβ, and RARγ) (Chambon, 1996), the signal mediated via RARγ plays a major role in chondro-osteogenesis (Williams et al., 2009; Shimono et al., 2011; Uchibe et al., 2017). Systemic administration of selective RARγ agonists effectively blocks heterotopic ossification in BMP-induced ectopic bone formation model and fibrodysplasia ossificans progressive (FOP) model animals (Shimono et al., 2011; Chakkalakal et al., 2016). Conversely, administration of RARγ antagonists enhanced cartilage tissue formation and ectopic bone, respectively (Uchibe et al., 2017). Neither selective RARγ agonist nor antagonist exhibit their effects in RARγ null mice.

In this study, we hypothesized that local treatment of RARγ antagonists potentiates bone inductive activity of BMP-2. We synthesized 7C (described in WO 2005/066115 A2) and loaded into polylactide-nanoparticles (PLA-NPs). This 7C contains 7a and 7a enantiomer, where 7a enhanced BMP-2 induced ectopic bone formation (Uchibe et al., 2017). The 7C is an inverse agonist that activates repressor function of RARγ but does not competitively inhibit the binding of retinoic acid with RARγ. Therefore, 7C does not show agonistic activity even when applied at high concentrations, while other RARγ antagonists may show agonist activity at high doses. We evaluated the therapeutic potential of 7C as an enhancer of BMP-2 in a mouse ectopic bone formation model and characterized its action in the process of endochondral ossification.

## 2 Materials and Methods

### 2.1 Retinoids and Nanoparticles

NRX 204647 (selective RARγ agonist, CAS 1351452-80-6) and 7C (selective RARγ antagonist, described in WO 2005/066115 A2) were synthesized by Atomax Chemicals (Shenzhen, China). Structures of those compounds are shown in Supplementary Figure S1. CD2665 (RARγ antagonists, CAS 170355-78-9) and CD1530 (RARγ agonist, CAS:107,430-66-0) were purchased from Cayman Chemical Company (Ann Arbor, MI, United States). For the *in vitro* study, synthetic retinoids were dissolved in DMSO to make a 1 mM stock solution, which was further diluted with 200 proof ethanol. For the animal study, NRX204647 or 7C were loaded into polylactide-nanoparticles (PLA-NPs) for sustained local delivery. Retinoid-loaded NPs were formulated using a modification of the emulsification-solvent evaporation method with albumin as a colloidal stabilizer (Chorny et al., 2010; Chorny et al., 2012). Synthetic retinoid loaded PLA-NPs are biodegradable and release the active drug for a prolonged time period (Shield et al., 2020).

### 2.2 Mouse Ectopic Bone Formation Model

#### 2.2.1 Experimental Design

All animal experiments were approved by the Animal Experimental Committee of our institution. Six-week-old C57BL/6J mice were purchased from Charles River Laboratories Japan, Inc. (Kanagawa, Japan). Briefly, collagen sponge discs (pellets) with 5.0 mm of diameter were prepared by punching out absorbable collagen sponges (CollaTape; Zimmer Dental, Carlsbad, CA, United States). In total, 20 μl of PBS containing 1.5 μg of recombinant human (rh) BMP-2 and NPs (blank-NP, 7C-NP, or NRX204647-NP) was soaked into the pellets. The pellets were implanted underneath the left and right dorsal fascia. The mice were divided into six groups: 1) blank-NP (control, *n* = 25), 2) 0.3 μg of 7C-NP (7C-0.3, *n* = 17), 3) 1.0 μg of 7C-NP (7C-1.0, *n* = 17), 4) 5.0 μg of 7C-NP (7C-5.0, *n* = 25), 5) 1.0 μg of NRX204647-NP (NRX-1.0, *n* = 7), and 6) 5.0 μg of NRX204647-NP (NRX-5.0, *n* = 7). Unless specified, the 7C group refers to the 7C-5.0 group. The pellets were explanted 14 days after surgery followed by micro-CT imaging and histological analysis. In the control and 7C-5.0 groups, the pellets were also explanted at Day 7 for histological analysis and quantification of sulfated glycosaminoglycan (sGAG) (*n* = 8 per group).

#### 2.2.2 Micro-CT Analysis

The pellets were explanted at Day 14 and scanned using the *ex vivo* high-resolution micro-CT (SkyScan 1272; Bruker, Billerica, MA, United States) with a source voltage of 80 kV, source current of 125 μA, and pixel size of 10 μm. Then, they were analyzed using micro-CT software (CT-Analyzer, Bruker).

#### 2.2.3 Histological Analyses and Immunohistochemistry

The dissected and formalin-fixed tissue samples were decalcified in 10% ethylenediaminetetraacetic acid, dehydrated via an ethanol series, embedded in paraffin wax, and serially sectioned at 3-μm thickness. Hematoxylin and eosin (H&E), safranin O staining, and immunohistochemical staining were performed. The list of antibodies and their detection conditions are listed in Supplementary Table S1. The antibodies for osteocalcin (OCN), p-Smad1, p-Smad2, Sox9, and CD163 were visualized using Histofine^®^ Simple Stain MAX PO (Nichirei Bioscience, Tokyo, Japan) and Simple Stain DAB Solution (Nichirei Bioscience). The antibodies for αSMA and OCN were stained with Alexa Fluor Plus 488–conjugated goat anti-rabbit secondary antibody (A32731, 1:1000; Thermo Fisher Scientific, Waltham, MA, United States) and Alexa Fluor 555-conjugated goat anti-rat secondary antibody (A21434, 1:1000; Thermo Fisher Scientific) for 1 h, followed by nuclear staining with 4′,6-diamidino-2-phenylindole solution (Dojindo Laboratories, Kumamoto, Japan) and mounting with Prolong Diamond Antifade Mountant (Thermo Fisher Scientific). BZ-X700 All-in-one Fluorescence Microscope (Keyence Corp., Osaka, Japan) was used to observe and capture the fluorescence images. To evaluate cartilage tissues, the area stained by safranin O staining was quantified using ImageJ (version 1.52q; U. S. National Institutes of Health; https://imagej.nih.gov/ij/) (Schneider et al., 2012). The bone shell thickness of the BMP-2 pellets was measured at randomly selected sites (eight sites/pellets). The immunopositive cells for p-Smad1, p-Smad2, and Sox9 were counted within cartilaginous tissue in BMP-2 pellets (300 × 300 μm, 2 fields/pellet), as previously described (Uchibe et al., 2017).

#### 2.2.4 Sulfated Glycosaminoglycans in BMP Pellets

The BMP-2 pellets were digested with 0.05% papain (Sigma-Aldrich, St. Louis, MO, United States) for 18 h at 65°C. Then, the amounts of the sulfated glycosaminoglycans (sGAGs) were measured using a dimethylmethylene blue dye-binding assay (Blyscan™ Glycosaminoglycan Assay Kit, Biocolor, Westbury, NY, United States).

#### 2.2.5 Evaluation of Inflammatory Response

The BMP-2 pellets, 1.5 μg rhBMP-2 (BMP 1.5 μg, *n* = 6), 2.25 μg rhBMP-2 (BMP 2.25 μg, *n* = 4), 3.0 μg rhBMP-2 (BMP 3.0 μg, *n* = 4), 1.5 μg rhBMP-2, and 5.0 μg of 7C-NP (BMP 1.5 μg + 7C, *n* = 6) were harvested on Day 7 and stained with H&E. The pellets without rhBMP-2, 5.0 μg of blank-NP (Blank, *n* = 6), 1.0 μg or 5.0 μg of 7C-NP (7C, *n* = 6 each) were also harvested. The area of the inflammatory zones, defined by infiltration of inflammatory cells such as histiocytes and fibroblasts (Lee et al., 2012; Huang et al., 2014), were measured using ImageJ. In addition, mRNAs were prepared from the pellets of the BMP 1.5 μg and BMP 1.5 μg + 7C groups (*n* = 12 per each group), and the gene expression levels of inflammatory cytokines were measured by real-time polymerase chain reaction.

### 2.3 *In Vitro* Experiment

#### 2.3.1 *In Vitro* Chondrogenic Differentiation

ATDC5 cells (Riken Cell Bank, Tsukuba, Japan) and mesenchymal stem cells (MSCs; Cyagen, Guangdong, China) were cultured as micromass cultures as described previously (Seriwatanachai et al., 2012). Briefly, ATDC5 cells and MSCs were spotted at 1 × 10^5^ cells in 10 μl and cultured in chondrogenic medium [DMEM containing 1% ITS + Premix Universal Supplement (Corning Inc., NY, United States), 50 μg/ml ascorbic acid (Sigma-Aldrich), 40 μg/ml L-proline (Wako, Osaka, Japan), 100 nM dexamethasone (Sigma-Aldrich), 10 ng/ml transforming growth factor β3 (PeproTech, Rocky Hill, NJ, United States), and 1% antibiotic-antimitotic solution (Sigma-Aldrich)]. The cultures were treated with 20 ng/ml of rhBMP-2 and DMSO or retinoid reagents (50 nM 7C or 100 nM NRX 204647) for 6 days, and fixed with 4% paraformaldehyde, stained with Alcian blue (pH 1.0). The staining intensity was quantified using ImageJ (Schneider et al., 2012).

#### 2.3.2 Immunoblotting

ATDC5 cells were grown to approximately 70% confluence in six-well plates in Dulbecco’s modified Eagle’s medium/Ham’s nutrient mixture F-12 containing 5% fetal bovine serum (FBS). Next, serum deprivation (0.3% FBS) was performed for 16 h. Subsequently, cells were treated with 20 ng/ml of rhBMP-2 and 7C (0–0.2 μM), CD2665 (0–1.0 μM), NRX204647 (0–0.2 μM), or CD1530 (0–1.0 μM) for 45 min. The total cellular proteins were harvested in radioimmunoprecipitation assay buffer (Thermo Scientific) supplemented with 1% protease/phosphatase inhibitor cocktail (Cell Signaling Technology, Inc., Danvers, MA, United States). Then, the protein concentrations were measured using the bicinchoninic acid method. Cell proteins were separated into 4–12% Bis-Tris gels (Life Technologies) and were transferred to polyvinylidene difluoride membranes (Nippon Genetics, Tokyo, Japan). After blocking with 5% skim milk, membranes were incubated overnight at 4°C with dilutions of antibodies against phospho-Smad1 (5753, 1:1000, Cell Signaling Technology), and glyceraldehyde 3-phosphate dehydrogenase (GAPDH) (2118, 1:1000, Cell Signaling Technology), followed by incubation with a horseradish peroxidase-conjugated secondary antibody (7074, 1:1000, Cell Signaling Technology). The ECL plus Western Blotting Detection System kit (GE Healthcare, Chicago, IL, United States) was used to detect immunoreactive proteins.

#### 2.3.3 Real-Time Polymerase Chain Reaction

ATDC5 cells and MSCs were cultured in 24-well plates in chondrogenic medium supplemented with rhBMP-2 (20 ng/ml) and 7C (50 nM) or NRX (100 nM) for 5 days. Cells or BMP-2 pellets were homogenized in TRIzol Reagent (Invitrogen, Carlsbad, CA, United States). Total RNA was extracted using the Direct-zol RNA kit (Zymo Research, Orange, CA, United States) and was reverse-transcribed to cDNA using ReverTra Ace qPCR RT Master Mix (Toyobo, Osaka, Japan). Gene expression was measured using real-time PCR with SYBR green master mix (Applied Biosystems, Foster City, CA, United States) in the Step One Plus Real-Time PCR System (Applied Biosystems, Foster City, CA, United States). Target gene expression levels were normalized to those of GAPDH, and fold changes were calculated relative to the control group using the 2^−∆∆ Ct^ method. Supplementary Table S2 shows the primer sequences.

#### 2.3.4 Reporter Assay

Cignal Finder 45-pathway reporter array plate (CCA 901L; Qiagen, Germany) was used to profile the action of selective RARγ ligands on other signaling pathways. The details and further information about the array plate and experimental protocol are described in the handbook, which is downloadable from the manufacturer’s website (www.qiagen.com). In brief, we prepared a transfection reagent mixture for the entire plate and mixed the mixture with DNA (per well: 20 ng RARγ-pSG5 expression vector, 200 ng pathway specific firefly-luc reporter and 5 ng constitutively active Renilla-luc reporter). We then dispensed a single cell suspension of AD293 cells (TaKaRa Bio, Japan) at a density of 40,000 cells per well. On the next day, medium was replaced and the drug treatment started. The reporter array plate was subjected to dual luciferase assay 24 h after the drug treatment. Changes of pathway-specific activity was expressed by Log2 of fold changes of relative reporter activity of the drug treated wells over those of the control wells. Another transient transfection assay was carried out as described previously (Uchibe et al., 2017). 4xA1-p89-luc contains four tandem copies of Sox9 binding cartilage-specific enhancer of the mouse aggrecan gene connected to Col2a1 derived minimal promoter. This reporter plasmid was used to monitor activity of Sox9 and L-Sox5/Sox6 (Han and Lefebvre, 2008). Id1-luc (Katagiri et al., 2002) and RARE-luc reporter RARE-luc (retinoic acid response element reporter) (Hoffman et al., 2006) were used to monitor BMP-Smad signaling and retinoic acid receptor signaling, respectively.

### 2.4 Statistical Analysis

Two groups were compared using the unpaired Student’s *t*-test, and three or more groups with one-way analysis of variance (ANOVA), followed by the Bonferroni multiple comparison test. Data were expressed as mean ± standard deviation and were analyzed using GraphPad Prism 8.0. *p*-Values of <0.05 were considered statistically significant.

## 3 Result

### 3.1 A Mouse Model of Ectopic Bone Formation

**FIGURE 1:**
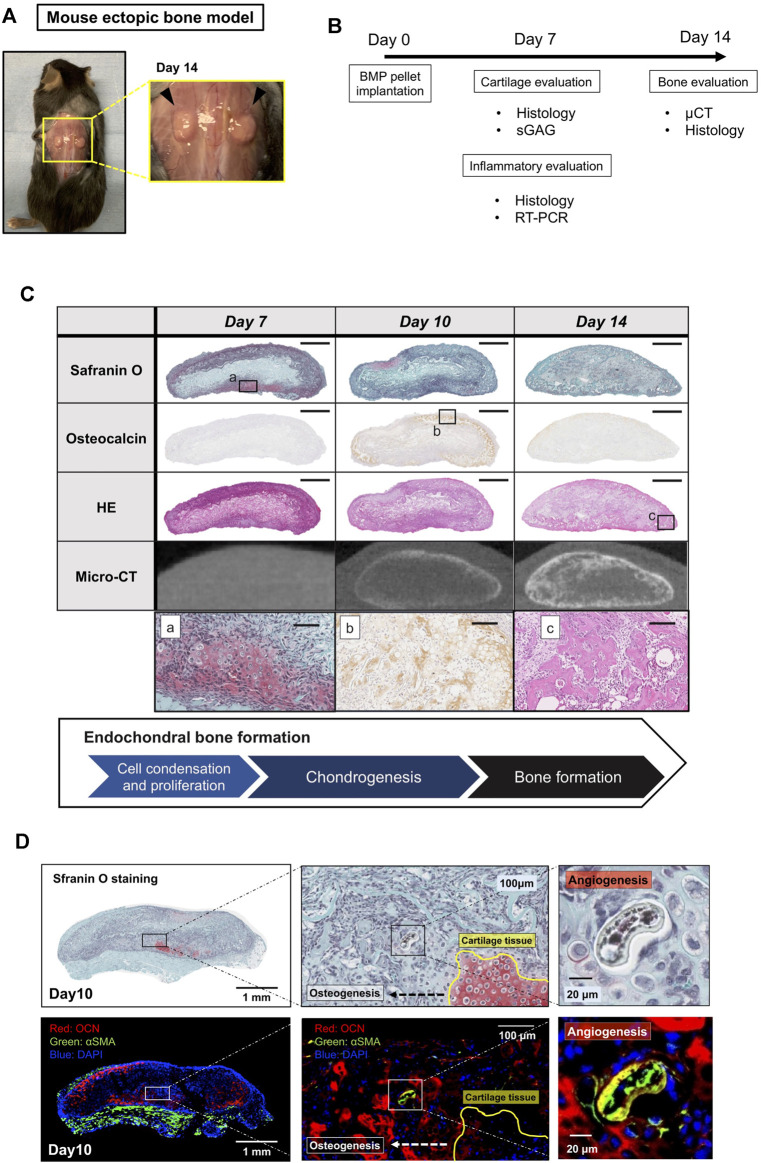
A mouse model of BMP-2-induced ectopic bone formation. **(A)** BMP-2 pellets 14 days after implantation. **(B)** The experiment outline. **(C)** Histology and μCT images of the BMP-2 pellets on Days 7, 10, and 14 (scale bar = 1 mm or 100 μm for whole view and the magnified view, respectively). **(D)** The BMP-2 pellet on Day 10. Cartilage tissue was replaced by new bone (OCN, red) and vasculature (αSMA, green).

We first characterize our ectopic bone formation model. The BMP-2 pellets implanted underneath the dorsal fascia were harvested on Day 7, 10, and 14 and were subjected to radiological, histochemical, and molecular biological assessments (Figures 1A,B). Figure 1C showed the endochondral bone formation process in the BMP-2 pellets on Days 7, 10, and 14. By Day 7, chondrocytes appeared around the pellet forming cartilaginous tissue. By Day 10, cartilage tissue began to replace bone tissue with signs of angiogenesis inside the pellets (Figure 1D). By Day 14, the majority of the pellets were composed of mature bone tissue. The results validated that this ectopic bone formation model was a reproducible model for ectopic bone formation and suitable to examine the drug effectiveness in a quantitative manner.

### 3.2 The Effects of 7C-NPs on Ectopic Bone Formation

**FIGURE 2:**
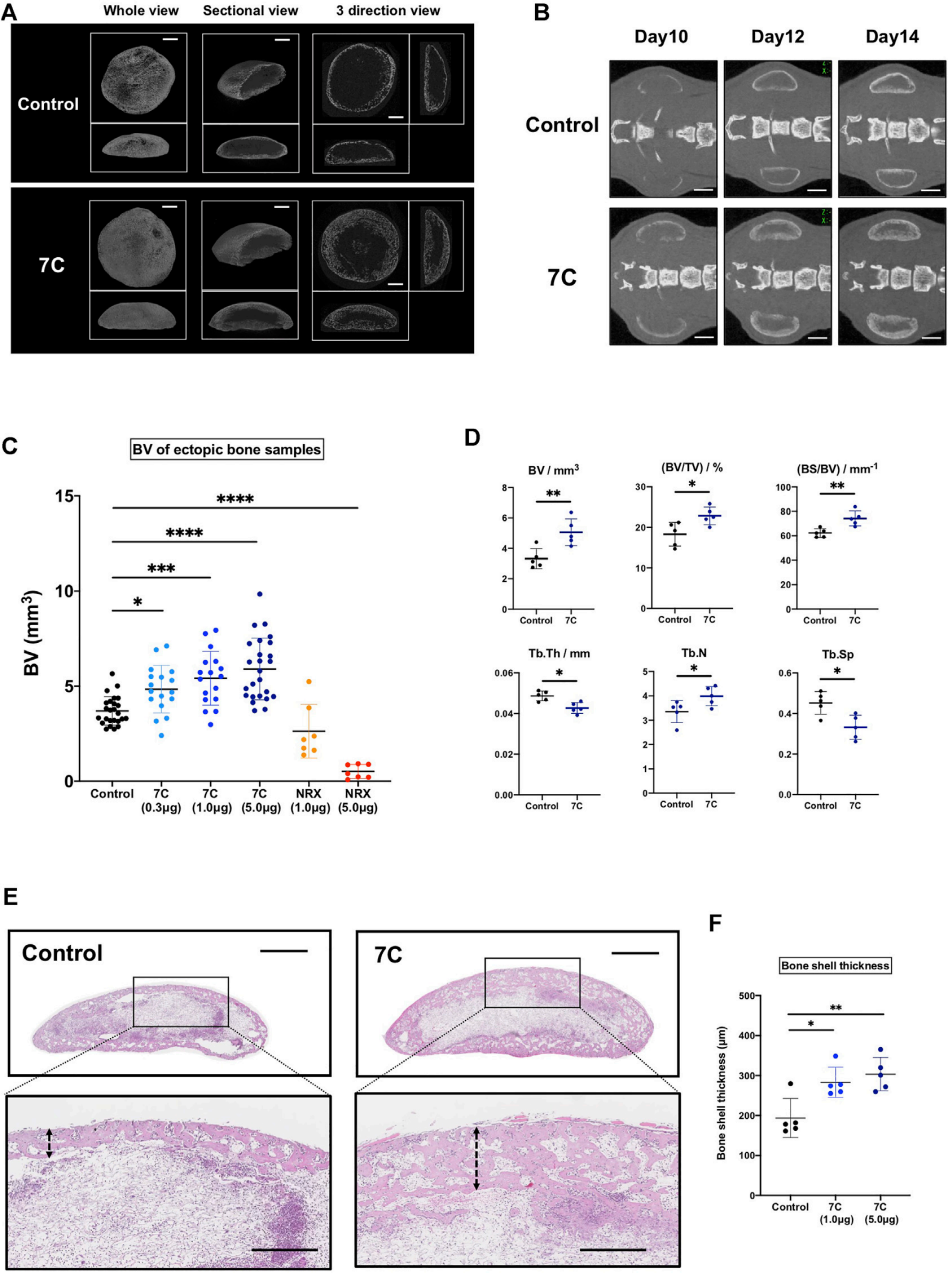
Analysis of bone induced in the BMP-2 pellets on Day 14. **(A)** *Ex vivo* 3D micro-CT images of the ectopic bone (scale bar = 1 mm). **(B)** *In vivo* micro-CT images of ectopic bones (scale bar = 2 mm). **(C)** The BV of the ectopic bone in the 7C and NRX groups. The BV was significantly higher in the 7C groups and lower in the NRX groups in a dose-dependent manner compared to the control value (control; *n* = 25, 7C-0.3; *n* = 17, 7C-1.0; *n* = 17, 7C-5.0; *n* = 25, NRX-1.0; *n* = 7, and NRX-5.0; *n* = 7; *p < 0.05, ***p < 0.001, and ****p < 0.0001). **(D)** Comparison of microstructural bone parameters between the control and 7C groups. *n* = 5, *p < 0.05, and **p < 0.01. **(E)** Histological sections (H&E staining) of BMP-2 pellets (whole view, scale bar = 1 mm; magnified view, scale bar = 400 μm). 7C group formed thicker bone shell. **(F)** Comparison of bone shell thickness. *n* = 5, *p < 0.05 and **p < 0.01.

We first tested whether local application of RARγ antagonists stimulated ectopic bone formation. We selected the 7C compounds for this purpose because 7C stimulates the ectopic bone formation via systemic administration (Uchibe et al., 2017) and has similar chemical properties in size, organophilicity and structure to those of NRX204647 (Supplementary Figure S1). The NRX204647 loaded PLA-NPs successfully inhibit chondrosarcoma growth with minimum local tissue damage (Shield et al., 2020). To examine the action of 7C-NPs on bone formation, 7C-NPs or blank-NPs were mixed into the BMP-2 pellets. Figures 2A,B show the representative *ex vivo* and *in vivo* CT images of the BMP-2 pellets with 7C-NPs or blank NPs**.** The BV of the pellets was significantly higher in the 7C group than that in the control group, and the effect of 7C-NPs was in a dose-dependent manner. In contrast, NRX204647-NPs reduced the bone volume (Figure 2C, NRX). The 7C-NP group formed more dense microstructural bone compared to the blank-NP group (Figure 2D, Control vs. 7C). The histology revealed that the 7C group had a thicker bone shell than the control group (Figures 2E,F).

### 3.3 The Effect of 7C on Cartilage Formation

**FIGURE 3:**
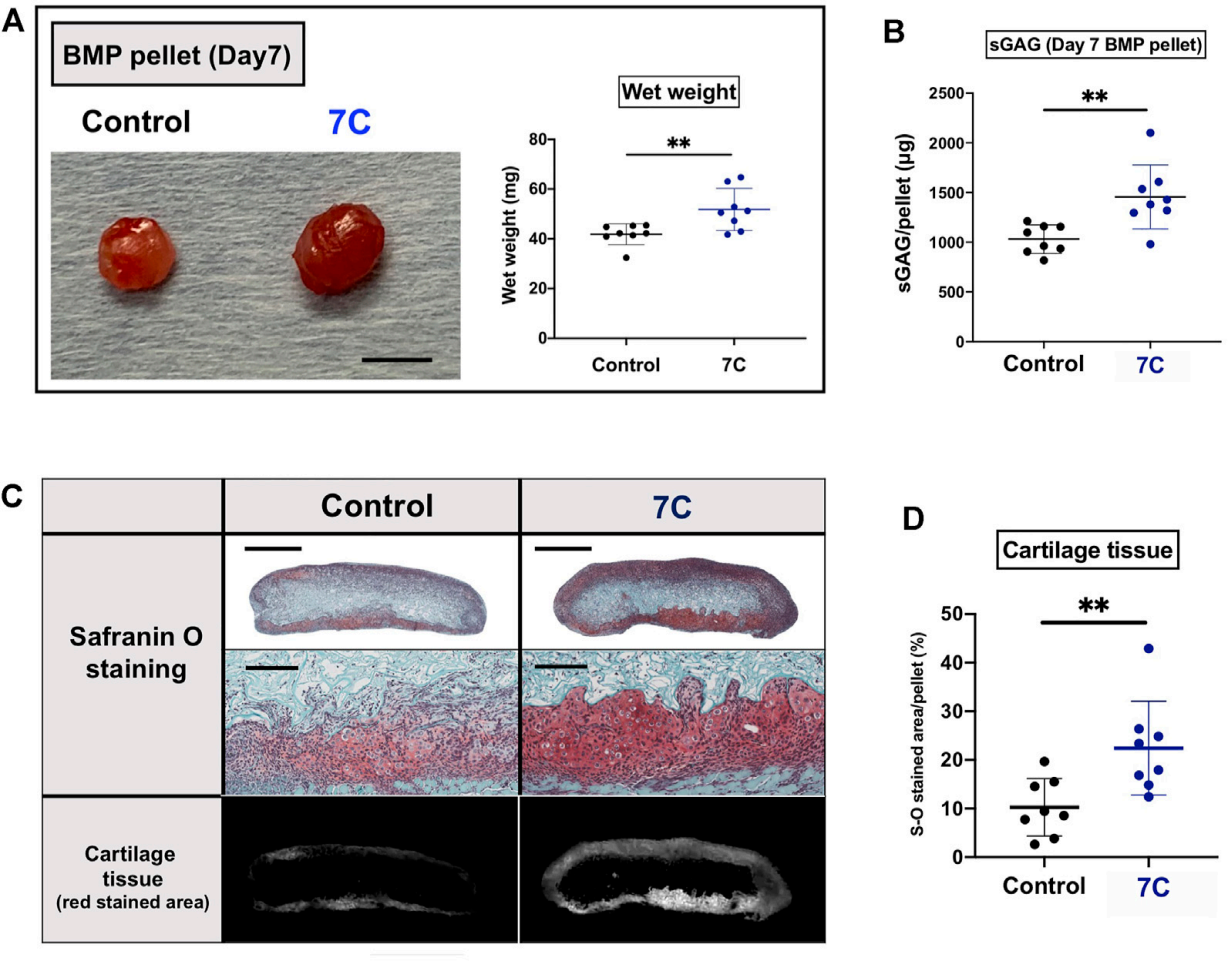
Evaluation of cartilage components in BMP pellets on Day 7. **(A)** Macroscopic appearance and wet weight of the BMP-2 pellets with 7C-NPs (7C) and blank-NP (Control) (scale bar = 5 mm). **(B)** The sGAG amount in the BMP-2 pellets. **(C)** Safranin O staining of the BMP-2 pellets (whole view, scale bar = 1 mm; magnified view, scale bar = 200 μm). **(D)** Comparison of cartilage tissue area. The red stained area was measured using ImageJ (version 1.52q, U. S. National Institutes of Health; https://imagej.nih.gov/ij/). *n* = 8 per group, **p < 0.01.

**FIGURE 4:**
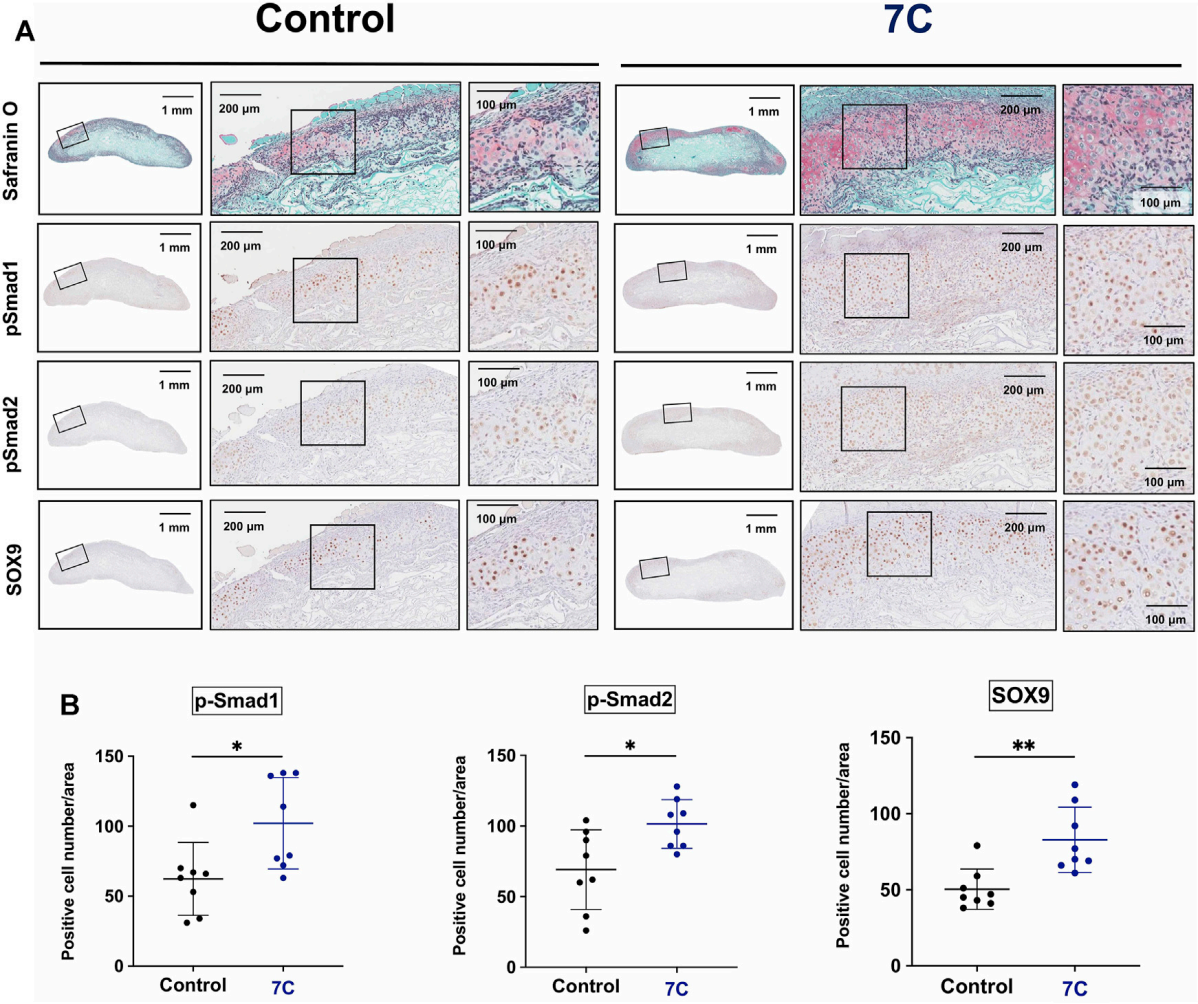
Immunohistochemical staining of BMP-2 pellets on Day 7. **(A)** p-Smad1, p-Smad2, and Sox9 positive cells in the cartilage tissue of the BMP-2 pellets with blank-NPs (Control) and 7C-NPs (7C). **(B)** Quantitative analysis of positive cells. The immunopositive cells for p-Smad1, p-Smad2, and Sox9 were counted only in cartilaginous tissue formed in BMP-2 pellets. *n* = 8 per group, *p < 0.05, and **p < 0.01.

To understand the action of 7C, cartilage formation was examined on Day 7 (Figure 1C). The average of the wet weight of BMP-2 pellets was significantly heavier, and the sGAG amounts were significantly higher in the 7C group than the control group (Figures 3A,B). The Safranin O-stained cartilage tissue area of the BMP-2 pellets was also larger in the 7C group than that in the control group (Figures 3C,D). These results suggest that 7C enhanced cartilage formation during the BMP-induced endochondral ossification. Activation of the BMP/Smad signaling is closely linked to cartilage formation (Hoffmann and Gross, 2001; Thielen et al., 2019). *In vivo* enhancement of BMP/Smad signaling pathway by 7C was investigated by immunohistochemical staining of p-Smad1. In the cartilage tissue of BMP-2 pellets on day 7, the number of p-Smad1-positive cells in the nucleus was observed to be higher in the 7C group than in the control group (Figures 4A,B). Higher positivity rates were also detected for p-Smad2 and Sox9 in the 7C group compared to the control group, suggesting that 7C stimulates the Smad2 pathway (Figures 4A,B). These analyses were performed only in cartilaginous tissue areas (not including bone tissue).

### 3.4 Analyses of Inflammation Around BMP Pellets

**FIGURE 5:**
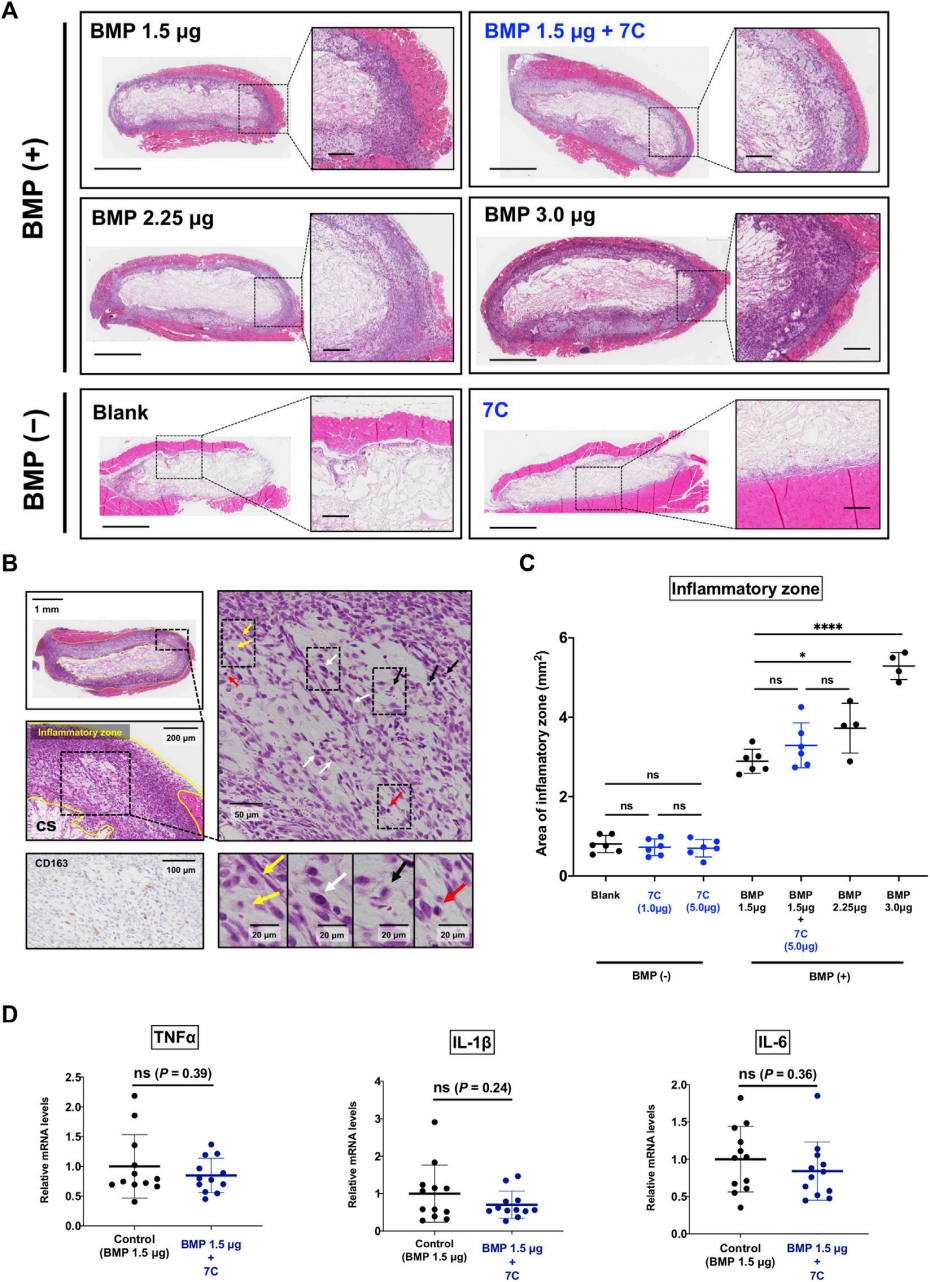
Effect of 7C on inflammatory response. **(A)** Representative microscopic images of the inflammatory zone stained with H&E in the pellets; the inflammatory zone expanded depending on the BMP amount, and the pellets without BMP (containing only blank-NP or 7C-NP) showed a small inflammatory zone. Whole view, scale bar = 1 mm; magnified view, scale bar = 200 μm. **(B)** Histological analysis of the inflammatory zone. The tissue (inflammatory zone) surrounding the collagen sponge (CS) was infiltrated with inflammatory cells such as macrophages (yellow arrow), fibroblasts (white arrow), leukocytes (black arrow) and lymphocytes (red arrow). Immunohistochemistry for CD163 that is positive in histiocytes. **(C)** The analysis of the inflammatory zone. *p < 0.05, ****p < 0.0001, and ns, not significant. **(D)** The mRNA expression levels of inflammatory cytokines (TNF-α, IL1-β, and IL-6) in the BMP-2 (1.5 μg) pellets with or without 7C-NPs. *n* = 12 per group. ns, not significant.

Previous reports have demonstrated that BMP-2 exaggerates inflammatory response, which may result in undesirable adverse events (Cahill et al., 2009; James et al., 2016; Zara et al., 2011). If 7C increases BMP-2-induced endochondral ossification solely by activating BMP signaling, we should see a more severe inflammatory reaction by applying 7C together with BMP-2. To elucidate this, we examined the histology of the pellets with 7C-NP or blank-NP in the presence or absence of BMP-2 (Figure 5A). The aggregation of inflammatory cells including histiocytes, granulocytes, and fibroblasts was observed at the margin of the pellet (inflammatory zone), as reported (Lee et al., 2012; Huang et al., 2014) (Figure 5B). We observed infiltration of inflammatory cells within the pellets and in the surrounding muscle tissue in all BMP-2 pellets. BMP-2 increased the area of inflammation in a dose-dependent manner (Figure 5C). In blank-NP and 7C-NP pellets without BMP-2, a thin line of inflammation was observed near the surface of the pellets. There was no noticeable difference in the size of inflammatory zones between blank-NP and 7C-NP pellets, indicating that 7C has no proinflammatory action (Figures 5A,C). The inflammatory zones of 7C-NP + 1.5 μg BMP-2 pellets were slightly larger than those of 1.5 μg BMP-2 only pellets, but the difference was not statistically significant. The expression levels of major inflammatory cytokines (TNF-α, IL1-β, and IL-6) were similar between 1.5 μg BMP-2 only and 7C-NP + 1.5 μg BMP-2 pellets, indicating that 7C-NP did not affect local inflammation caused by BMP-2 (Figure 5D).

### 3.5 Effects of RARγ Antagonists and Agonists on ATDC5 Cells

**FIGURE 6:**
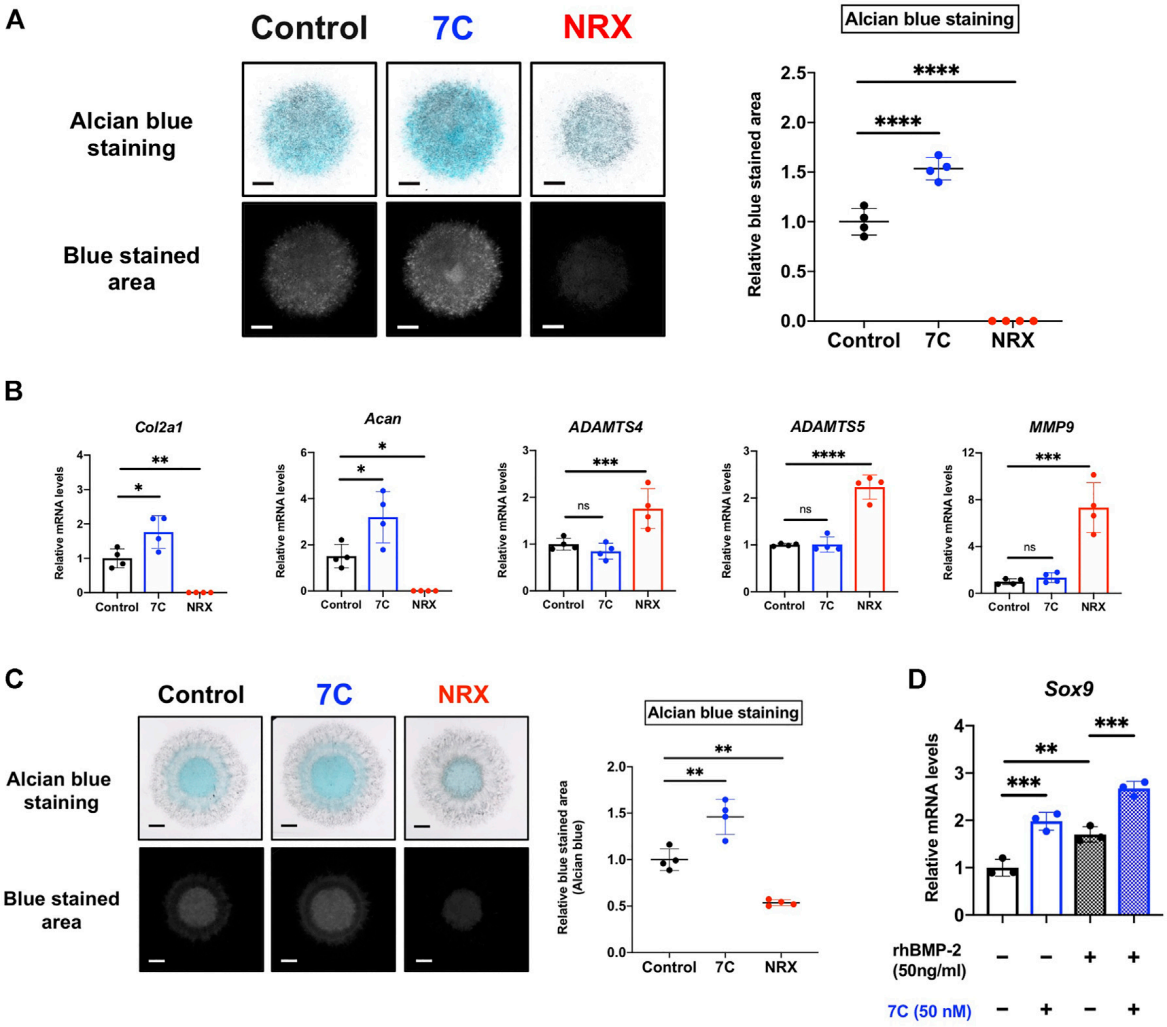
Effect of RARγ agonist/antagonist on ATDC5 cells and MSCs. **(A)** Alcian blue staining of ATDC5 cells in micromass (scale bar = 1 mm). The blue stained area was extracted using ImageJ (version 1.52q, U. S. National Institutes of Health; https://imagej.nih.gov/ij/) (*n* = 4 per group). **(B)** Quantitative RT-PCR analysis of ATDC5 cells cultured in chondrogenic medium supplemented with BMP-2 (20 ng/ml) and 7C (50 nM) or NRX (100 nM) (*n* = 4 per group). **(C)** Alcian blue staining of MSCs in micromass (scale bar = 1 mm). The blue stained area was extracted using ImageJ (*n* = 4 per group). **(D)** The gene expression level of *Sox9* in MSCs cultured in chondrogenic medium. *Sox9* expression was upregulated by 7C, even in the absence of BMP-2 (*n* = 3 per group). *p < 0.05, **p < 0.01, ***p < 0.001 and ****p < 0.0001 and ns, not significant.

**FIGURE 7:**
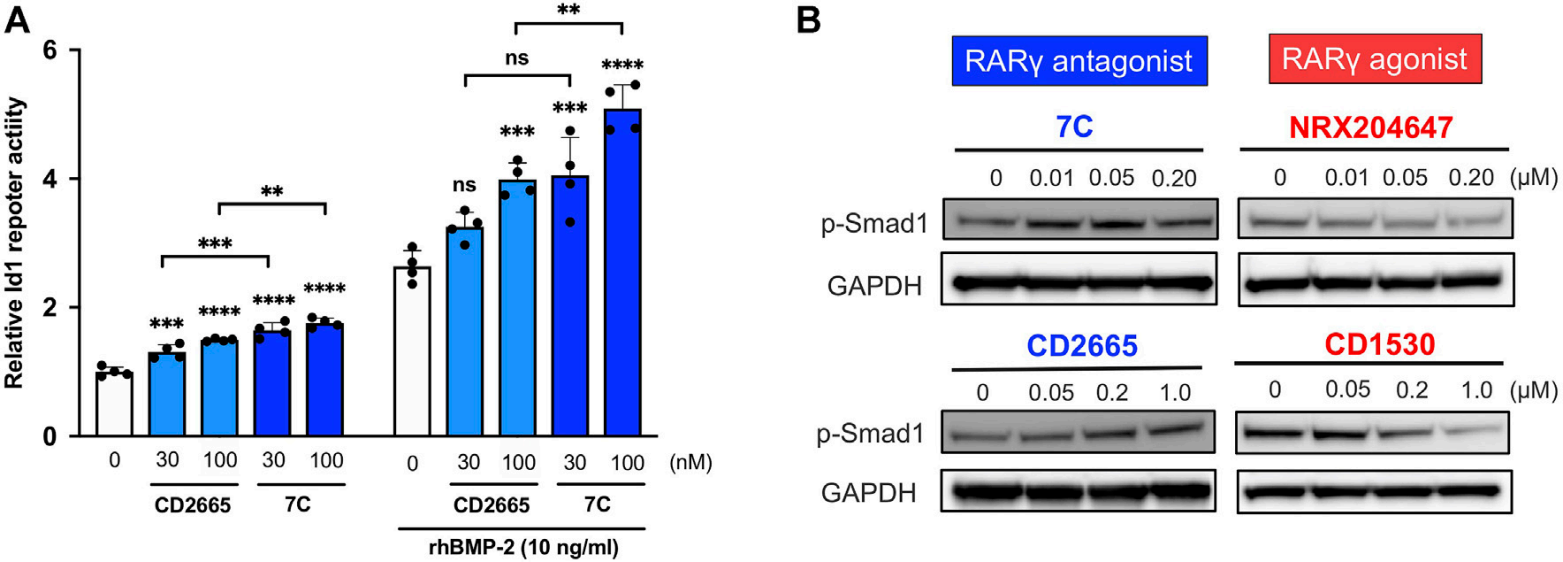
Effect of RARγ antagonists and agonists on BMP signaling. **(A)** Id1-luc reporter activity in ATDC5 cells treated with RARγ antagonists (CD2665 and 7C) with or without 10 ng/ml rhBMP-2. *n* = 4 per group. **p < 0.01, ***p < 0.001, ****p < 0.0001, and ns, not significant. **(B)** Immunoblotting analysis with p-Smad1 antibody. The ATDC5 cells were treated with BMP-2 and increasing concentrations of RARγ antagonists (CD2665 and 7C) and RARγ agonists (NRX204647 and CD1530). The cell lysates were prepared 45 min after treatment and subjected to immunoblot analysis for p-Smad1 and GAPDH.

To study cellular actions of RARγ antagonists, we performed *in vitro* studies using ATDC5 cells. ATDC5 cells can exhibit similar activity to chondrogenic differentiation—initiation of cartilage matrix synthesis, accumulation of cartilage matrix, and terminal differentiation, corresponding to the early, mid, and late phases of chondrocyte differentiation seen during endochondral bone formation *in vivo* (Shukunami et al., 1997). To study the actions of 7C on chondrogenic differentiation, we used this ATCD5 culture system. 7C treatment enhanced sulfated proteoglycan accumulation in micromass culture and increased the gene expression of *Col2a1* and *Acan* (Figures 6A,B). Conversely, treatment with RARγ agonist, NRX204647 suppressed the proteoglycan accumulation and gene expression of *Col2a1* and *Acan*, and further upregulated the gene expression of matrix degradation enzymes, *Adamts4, Adamts5* and *Mmp9* (Figures 6A,B). We also examined the effect on the BMP-Smad signaling pathway. The RARγ antagonists 7C and CD2665 moderately stimulated BMP/Smad signaling and also enhanced the action of BMP-2, as determined by the Id-luc reporter assay (Figure 7A). The immunoblot analysis with anti-pSmad1 antibody revealed that the phosphorylation of Smad1 was enhanced by RARγ antagonists (7C and CD2665) and suppressed by RARγ agonists (NRX204647 and CD1530) (Figure 7B).

### 3.6 Effects of RARγ Antagonists and Agonists on MSCs

Condensations of MSCs and their chondrogenic differentiation play a key role in endochondral bone formation (Kronenberg, 2003; Scotti et al., 2010; Freeman et al., 2015; Knuth et al., 2017). The actions of RARγ antagonists and agonists on chondrogenesis were also examined in the MSC cultures. As seen in the ATDC5 culture, 7C also enhanced the proteoglycan accumulation in MSCs in micromass while NRX204647 suppressed it (Figure 6C). In addition, 7C upregulated the expression of *Sox9*, a master regulator of chondrogenic differentiation, with or without BMP-2, indicating that 7C might enhance chondrogenic differentiation of MSCs even in the absence of BMP-2 (Figure 6D). To complement this work, we performed reporter assays with 4xA1-p89-luc (repoter for Sox9) and Id1-luc. RARγ antagonists enhanced the BMP-2 stimulatory action of the aggrecan promoter activity and stimulated Id1-luc activity (Supplementary Figures S2A,B). The retinoid reporter assay confirmed inhibitory action of RARγ antagonists on retinoid signaling (Supplementary Figure S2C).

## 4 Discussion

In this study, we demonstrated that in the mouse ectopic bone formation model, the co-administration of 7C-NP with BMP-2 increased the volume of newly formed bone after increased cartilage tissue formation (Figure 2). When BMP is applied locally to induce bone, it causes inflammation. Indeed, the size of the inflammatory zone was enlarged accordingly with increasing dosage of BMP-2 (Figure 5). Interestingly, 7C has no proinflammatory effect by itself and promoted BMP-2-induced bone formation without exaggerating BMP-2 induced local inflammation (Figure 5). Current problems of the BMP-based bone regeneration therapy include the high cost of the drugs and their adverse effects such as tissue edema and increased bone resorption, mainly due to local inflammation (James et al., 2016). 7C might solve such problems because BMP combination with 7C enhances bone induction with lower doses of BMPs, which may reduce drug costs and adverse effects. However, further studies, including histological and molecular assessments to elucidate the cellular and molecular effects of 7C and testing for efficacy in larger animal models should be conducted to conclude the therapeutic efficacy of this approach.

One possible mechanism of the enhancement of BMP bone-inducing action by 7C is the enhancement of BMP-Smad signaling activity on chondrogenesis. It has been reported that RA promotes the degradation of p-Smad1 (Sheng et al., 2010), and that selective RARγ agonists strongly reduce pSmad1/5/8 protein levels (Shimono et al., 2011). In line with those previous works, we confirmed that the selective RARγ antagonist 7C stimulated canonical BMP-Smad signaling *in vivo* and *in vitro* (Figures 4, 7). 7C did not significantly enhance the BMP-2-induced inflammatory response although it marginally widened the inflammatory zone in the presence of BMP-2. In contrast, 7C strongly stimulated BMP-2 induced bone formation in a dose-dependent manner. Therefore, it is unlikely that the stimulatory effect of 7C on bone formation is solely dependent on BMP-Smad signaling. The slight enlargement of the inflammatory zone by 7C is an acceptable reaction, given the potentiating effect of 7C on bone formation. 7C may stimulate non-canonical BMP signaling, which stimulates ectopic cartilage formation (Jin et al., 2006; Nepal et al., 2013). Additionally, stimulation of inflammation by BMP-2 may be dominantly mediated by the Smad signaling pathway. Notably, RARγ is reported to be a positive regulator of the inflammatory cytokine production of macrophages and CD8^+^ T cells (Dzhagalov et al., 2007; Gordy et al., 2009; Duong and Rochette-Egly, 2011). These reports suggest that RARγ antagonists may possess anti-inflammatory effects. Rather than increasing the BMP dose, co-administration of 7C enables efficient bone regeneration without an accompanying severe inflammatory response. However, the effect of 7C alone on the local inflammatory response should be pursued in the future.

Inhibition of RARγ signaling by 7C could stimulate chondrogenesis of mesenchymal cells in a BMP-independent manner after the mesenchymal cells are primed by exposure to BMP. The 7C used in this study are one of the most potent and highly selective RARγ antagonists. However, it should be noted that the biological effects of such compounds may be the sum of the RARγ-mediated on-target effects and compound specific off-target effects that are caused by interactions with other molecules. Indeed, we found that various intercellular pathways were affected by 7C and NRX204647 using the Cignal Finder 45-pathway reporter array plate (Supplementary Figure S3). 7C and NRX204647 predominantly demonstrated opposite effects on the affected pathways, suggesting that actions on those pathways were likely mediated by RARγ. Interestingly, NRX204647 suppressed cAMP-PKA-CREB, HIF1a, and TGFβ signaling pathways, whereas 7C enhanced them. These pathways are known to be involved in cartilage formation (Rosengart et al., 1975; Kosher and Savage, 1980; Seyedin et al., 1986; Johnstone et al., 1998; Nöth et al., 2002; Schipani, 2006; Yokoyama et al., 2015). The results are consistent with the immunohistochemistry results of BMP-2 pellets combined with 7C, which show a larger positive ratio of p-Smad2 and Sox9 positive cells, because p-Smad2 plays an important role in TGFβ signaling, and Sox9, a master regulator of chondrocytes, is closely regulated by cAMP-PKA-CREB and HIF1a (Amarilio et al., 2007; Zhao et al., 2009; Fernández-Torres et al., 2017). Taken together, the pro-chondrogenic effects via multiple signaling pathways, in addition to the mild enhancement of BMP signaling, may play roles in enhancing BMP-2 induced bone formation by 7C. Contribution of those signaling pathways in cartilage formation should be future studied. In addition, some off-target effects may be involved.

Because the 7C-NPs enable extended release of 7C, 7C could regulate bone formation and maintenance. Analysis of ectopic bone by micro-CT showed an increase in bone volume in the 7C + BMP-administered group compared with that of the BMP alone group. In addition, the thickness of the ectopic bone shell was markedly increased. To date, many animal studies have shown that administration of retinoic acid or its precursor leads to bone loss (Lind et al., 2013; Green et al., 2016). The mechanism has not been understood well, but it has been reported that retinoids promote osteoclastgenesis indirectly via other retinoid target cells such as BMSC (Conaway et al., 2011; Green et al., 2018). On the other hand, global knock down or Prrx-1Cre mediated limb specific ablation of RARγ gene results in bone loss (Green et al., 2018), importantly suggesting that RARγ is required for normal skeletal formation and homeostasis and that the non-liganded RARγ functions as a repressor of both. Selective RARγ antagonist 7C is classified as an inverse agonist (Klein et al., 1996; Nagpal and Chandraratna, 2000). It binds to RARγ with high affinity, stabilizes the heterodimer structure with RXR, and facilitates recruitment of co-repressors. Thus, enhancing the repressor function of RARγ might be an additional mechanism by which 7C increased bone formation.

In the animal experiment conducted, we chose the PLA-nanoparticle as the vehicle to locally deliver 7C in a sustained manner. The reason is that PLA-NP is a widely used drug carrier and is FDA approved. In addition, by applying the drug locally, we could minimize systemic side-effects. Furthermore, we recently formulated NRX204647-loaded PLA-NP which successfully inhibited chondrosarcoma growth with minimum local tissue damage (Shield et al., 2020). Since the chemical properties of NRX204647 and 7C are quite similar in size, organophilicity, and structure (Supplementary Figure S1), we predicted that PLA-NP is a suitable drug vehicle for our study. In fact, enhancement of BMP-induced bone formation by 7C was quite reproducible.

Before translation to clinical work, multiple studies must be conducted. Thorough investigation of drug safety, especially regarding local toxicity and systemic effects (liver and kidney function), is needed. In addition, it is important to prove its effectiveness in more clinically relevant experimental systems such as large bone defect, fracture, and spinal fusion. We are currently testing 7C in a rat spinal fusion model. We hope the experiment further validates the therapeutic value of the 7C for BMP-based bone regeneration. This study supports future studies on the cost, effectiveness, and safety of BMP-bases therapies.

## 5 Conclusion

Novel local drugs, specifically the PLA-NPs loaded with the synthetic selective RARγ antagonist, 7C, efficiently promoted BMP-2 induced endochondral bone formation. This establishes the potential of local co-administration of 7C-NPs and BMP-2 and its potential to develop a novel bone regeneration therapy by enhancing both bone quantity and quality while minimizing BMP-2-related adverse events.

## Data Availability

The original contributions presented in the study are included in the article/Supplementary Material, further inquiries can be directed to the corresponding authors.

## References

[B1] Amarilio R. Viukov S. V. Sharir A. Eshkar-Oren I. Johnson R. S. Zelzer E. (2007). HIF1α Regulation of Sox9 Is Necessary to Maintain Differentiation of Hypoxic Prechondrogenic Cells during Early Skeletogenesis. Development (Cambridge, England) 134 (21), 3917–3928. 10.1242/dev.008441 17913788

[B2] Betz R. R. (2002). Limitations of Autograft and Allograft: New Synthetic Solutions. Orthopedics 25 (5 Suppl. l), s561–70. 10.3928/0147-7447-20020502-04 12038843

[B3] Burkus J. K. Transfeldt E. E. Kitchel S. H. Watkins R. G. Balderston R. A. (2002). Clinical and Radiographic Outcomes of Anterior Lumbar Interbody Fusion Using Recombinant Human Bone Morphogenetic Protein-2. Spine 27 (21), 2396–2408. 10.1097/00007632-200211010-00015 12438990

[B4] Cahill K. S. Chi J. H. Day A. Claus E. B. (2009). Prevalence, Complications, and Hospital Charges Associated with Use of Bone-Morphogenetic Proteins in Spinal Fusion Procedures. Jama 302 (1), 58–66. 10.1001/jama.2009.956 19567440

[B5] Chakkalakal S. A. Uchibe K. Convente M. R. Zhang D. Economides A. N. Kaplan F. S. (2016). Palovarotene Inhibits Heterotopic Ossification and Maintains Limb Mobility and Growth in Mice with the HumanACVR1R206HFibrodysplasia Ossificans Progressiva (FOP) Mutation. J. Bone Miner Res. 31 (9), 1666–1675. 10.1002/jbmr.2820 26896819 PMC4992469

[B6] Chambon P. (1996). A Decade of Molecular Biology of Retinoic Acid Receptors. FASEB j. 10 (9), 940–954. 10.1096/fasebj.10.9.8801176 8801176

[B7] Chorny M. Alferiev I. S. Fishbein I. Tengood J. E. Folchman-Wagner Z. Forbes S. P. (2012). Formulation and *In Vitro* Characterization of Composite Biodegradable Magnetic Nanoparticles for Magnetically Guided Cell Delivery. Pharm. Res. 29 (5), 1232–1241. 10.1007/s11095-012-0675-y 22274555 PMC3336034

[B8] Chorny M. Fishbein I. Yellen B. B. Alferiev I. S. Bakay M. Ganta S. (2010). Targeting Stents with Local Delivery of Paclitaxel-Loaded Magnetic Nanoparticles Using Uniform fields. Proc. Natl. Acad. Sci. 107 (18), 8346–8351. 10.1073/pnas.0909506107 20404175 PMC2889533

[B9] Conaway H. H. Pirhayati A. Persson E. Pettersson U. Svensson O. Lindholm C. (2011). Retinoids Stimulate Periosteal Bone Resorption by Enhancing the Protein RANKL, a Response Inhibited by Monomeric Glucocorticoid Receptor. J. Biol. Chem. 286 (36), 31425–31436. 10.1074/jbc.M111.247734 21715325 PMC3173101

[B10] Duong V. Rochette-Egly C. (2011). The Molecular Physiology of Nuclear Retinoic Acid Receptors. From Health to Disease. Biochim. Biophys. Acta (Bba) - Mol. Basis Dis. 1812 (8), 1023–1031. 10.1016/j.bbadis.2010.10.007 20970498

[B11] Dzhagalov I. Chambon P. He Y.-W. (2007). Regulation of CD8+T Lymphocyte Effector Function and Macrophage Inflammatory Cytokine Production by Retinoic Acid Receptor γ. J. Immunol. 178 (4), 2113–2121. 10.4049/jimmunol.178.4.2113 17277115

[B12] Fernández-Torres J. Zamudio-Cuevas Y. Martínez-Nava G. A. López-Reyes A. G. (2017). Hypoxia-Inducible Factors (HIFs) in the Articular Cartilage: a Systematic Review. Eur. Rev. Med. Pharmacol. Sci. 21 (12), 2800–2810. 28682438

[B13] Freeman F. E. Allen A. B. Stevens H. Y. Guldberg R. E. McNamara L. M. (2015). Effects of *In Vitro* Endochondral Priming and Pre-vascularisation of Human MSC Cellular Aggregates *In Vivo* . Stem Cell Res Ther 6, 218. 10.1186/s13287-015-0210-2 26541817 PMC4635553

[B14] Gordy C. Dzhagalov I. He Y.-W. (2009). Regulation of CD8+ T Cell Functions by RARγ. Semin. Immunol. 21 (1), 2–7. 10.1016/j.smim.2008.07.002 18715802 PMC2615478

[B15] Govender S. Csimma C. Genant H. K. Valentin-Opran A. Amit Y. Arbel R. (2002). Recombinant Human Bone Morphogenetic Protein-2 for Treatment of Open Tibial Fractures. The J. Bone Jt. Surgery-American Volume 84 (12), 2123–2134. 10.2106/00004623-200212000-00001 12473698

[B16] Green A. C. Martin T. J. Purton L. E. (2016). The Role of Vitamin A and Retinoic Acid Receptor Signaling in post-natal Maintenance of Bone. J. Steroid Biochem. Mol. Biol. 155 (Pt A), 135–146. 10.1016/j.jsbmb.2015.09.036 26435449

[B17] Green A. C. Rudolph-Stringer V. Straszkowski L. Tjin G. Crimeen-Irwin B. Walia M. (2018). Retinoic Acid Receptor γ Activity in Mesenchymal Stem Cells Regulates Endochondral Bone, Angiogenesis, and B Lymphopoiesis. J. Bone Miner Res. 33 (12), 2202–2213. 10.1002/jbmr.3558 30040873

[B18] Han Y. Lefebvre V. (2008). L-Sox5 and Sox6 Drive Expression of the Aggrecan Gene in Cartilage by Securing Binding of Sox9 to a Far-Upstream Enhancer. Mol. Cell Biol 28 (16), 4999–5013. 10.1128/mcb.00695-08 18559420 PMC2519711

[B19] Henning P. Conaway H. H. Lerner U. H. (2015). Retinoid Receptors in Bone and Their Role in Bone Remodeling. Front. Endocrinol. 6, 31. 10.3389/fendo.2015.00031 PMC4356160 25814978

[B20] Hoffman L. M. Garcha K. Karamboulas K. Cowan M. F. Drysdale L. M. Horton W. A. (2006). BMP Action in Skeletogenesis Involves Attenuation of Retinoid Signaling. J. Cel. Biol. 174 (1), 101–113. 10.1083/jcb.200604150 PMC2064168 16818722

[B21] Hoffmann A. Gross G. (2001). BMP Signaling Pathways in Cartilage and Bone Formation. Crit. Rev. Eukaryot. Gene Expr. 11 (1-3), 23–45. 10.1615/critreveukargeneexpr.v11.i1-3.20 11693963

[B22] Huang R.-L. Yuan Y. Tu J. Zou G.-M. Li Q. (2014). Exaggerated Inflammatory Environment Decreases BMP-2/acs-Induced Ectopic Bone Mass in a Rat Model: Implications for Clinical Use of BMP-2. Osteoarthritis and cartilage 22 (8), 1186–1196. 10.1016/j.joca.2014.06.017 24981632

[B23] James A. W. LaChaud G. Shen J. Asatrian G. Nguyen V. Zhang X. (2016). A Review of the Clinical Side Effects of Bone Morphogenetic Protein-2. Tissue Eng. B: Rev. 22 (4), 284–297. 10.1089/ten.teb.2015.0357 PMC4964756 26857241

[B24] Jin E. J. Lee S. Y. Choi Y. A. Jung J. C. Bang O. S. Kang S. S. (2006). BMP-2-enhanced Chondrogenesis Involves P38 MAPK-Mediated Down-Regulation of Wnt-7a Pathway. Mol. Cell 22 (3), 353–359. 17202865

[B25] Johnstone B. Hering T. M. Caplan A. I. Goldberg V. M. Yoo J. U. (1998). In VitroChondrogenesis of Bone Marrow-Derived Mesenchymal Progenitor Cells. Exp. Cel. Res. 238 (1), 265–272. 10.1006/excr.1997.3858 9457080

[B26] Katagiri T. Imada M. Yanai T. Suda T. Takahashi N. Kamijo R. (2002). Identification of a BMP-Responsive Element inId1, the Gene for Inhibition of Myogenesis. Genes Cell : devoted Mol. Cell. Mech. 7 (9), 949–960. 10.1046/j.1365-2443.2002.00573.x 12296825

[B27] Klein E. S. Pino M. E. Johnson A. T. Davies P. J. A. Nagpal S. Thacher S. M. (1996). Identification and Functional Separation of Retinoic Acid Receptor Neutral Antagonists and Inverse Agonists. J. Biol. Chem. 271 (37), 22692–22696. 10.1074/jbc.271.37.22692 8798442

[B28] Knuth C. Witte-Bouma J. Witte-Bouma J. Ridwan Y. Wolvius E. Farrell E. (2017). Mesenchymal Stem Cell-Mediated Endochondral Ossification Utilising Micropellets and Brief Chondrogenic Priming. eCM 34, 142–161. 10.22203/eCM.v034a10 28937176

[B29] Kosher R. A. Savage M. P. (1980). Studies on the Possible Role of Cyclic AMP in Limb Morphogenesis and Differentiation. J. Embryol. Exp. Morphol. 56, 91–105. 10.1242/dev.56.1.91 6249880

[B30] Kronenberg H. M. (2003). Developmental Regulation of the Growth Plate. Nature 423 (6937), 332–336. 10.1038/nature01657 12748651

[B31] Laurie S. W. S. Kaban L. B. Mulliken J. B. Murray J. E. (1984). Donor-site Morbidity after Harvesting Rib and Iliac Bone. Plast. Reconstr. Surg. 73 (6), 933–938. 10.1097/00006534-198406000-00014 6374708

[B32] Lee K.-B. Taghavi C. E. Murray S. S. Song K.-J. Keorochana G. Wang J. C. (2012). BMP Induced Inflammation: a Comparison of rhBMP-7 and rhBMP-2. J. Orthop. Res.official Publication Orthopaedic Res. Soc. 30 (12), 1985–1994. 10.1002/jor.22160 22674456

[B33] Lind T. Sundqvist A. Hu L. Pejler G. Andersson G. Jacobson A. (2013). Vitamin a Is a Negative Regulator of Osteoblast Mineralization. PloS one 8 (12), e82388. 10.1371/journal.pone.0082388 24340023 PMC3858291

[B34] Lo K. W.-H. Ulery B. D. Ashe K. M. Laurencin C. T. (2012). Studies of Bone Morphogenetic Protein-Based Surgical Repair. Adv. Drug Deliv. Rev. 64 (12), 1277–1291. 10.1016/j.addr.2012.03.014 22512928 PMC3401330

[B35] Nagpal S. Chandraratna R. (2000). Recent Developments in Receptor-Selective Retinoids. Cpd 6 (9), 919–931. 10.2174/1381612003400146 10828316

[B36] Nepal M. Li L. Cho H. K. Park J. K. Soh Y. (2013). Kaempferol Induces Chondrogenesis in ATDC5 Cells through Activation of ERK/BMP-2 Signaling Pathway. Food Chem. Toxicol. 62, 238–245. 10.1016/j.fct.2013.08.034 23989061

[B37] Nöth U. Osyczka A. M. Tuli R. Hickok N. J. Danielson K. G. Tuan R. S. (2002). Multilineage Mesenchymal Differentiation Potential of Human Trabecular Bone-Derived Cells. J. Orthop. Res. 20 (5), 1060–1069. 10.1016/s0736-0266(02)00018-9 12382974

[B38] Rosengart R. Fishbein M. Emmanouilides G. C. (1975). Progressive Pulmonary Vascular Disease after Surgical Correction (Mustard Procedure) of Transposition of Great Arteries with Intact Ventricular Septum. Am. J. Cardiol. 35 (1), 107–111. 10.1016/0002-9149(75)90567-6 1109240

[B39] Schipani E. (2006). Hypoxia and HIF-1 in Chondrogenesis. Ann. N Y Acad. Sci. 1068, 66–73. 10.1196/annals.1346.009 16831906

[B40] Schneider C. A. Rasband W. S. Eliceiri K. W. (2012). NIH Image to ImageJ: 25 Years of Image Analysis. Nat. Methods 9 (7), 671–675. 10.1038/nmeth.2089 22930834 PMC5554542

[B41] Scotti C. Tonnarelli B. Papadimitropoulos A. Scherberich A. Schaeren S. Schauerte A. (2010). Recapitulation of Endochondral Bone Formation Using Human Adult Mesenchymal Stem Cells as a Paradigm for Developmental Engineering. Proc. Natl. Acad. Sci. 107 (16), 7251–7256. 10.1073/pnas.1000302107 20406908 PMC2867676

[B42] Seriwatanachai D. Krishnamra N. Charoenphandhu N. (2012). Chondroregulatory Action of Prolactin on Proliferation and Differentiation of Mouse Chondrogenic ATDC5 Cells in 3-dimensional Micromass Cultures. Biochem. biophysical Res. Commun. 420 (1), 108–113. 10.1016/j.bbrc.2012.02.123 22405773

[B43] Seyedin S. M. Thompson A. Y. Bentz H. Rosen D. M. McPherson J. M. Conti A. (1986). Cartilage-inducing Factor-A. Apparent Identity to Transforming Growth Factor-Beta. J. Biol. Chem. 261 (13), 5693–5695. 10.1016/s0021-9258(17)38436-3 3754555

[B44] Sheng N. Xie Z. Wang C. Bai G. Zhang K. Zhu Q. (2010). Retinoic Acid Regulates Bone Morphogenic Protein Signal Duration by Promoting the Degradation of Phosphorylated Smad1. Proc. Natl. Acad. Sci. 107 (44), 18886–18891. 10.1073/pnas.1009244107 20956305 PMC2973900

[B45] Shield W. P. 3rd Cellini A. Tian H. Wilson K. Dan Y. Abzug J. M. (2020). Selective Agonists of Nuclear Retinoic Acid Receptor Gamma Inhibit Growth of HCS‐2/8 Chondrosarcoma Cells. J. Orthop. Res. 38 (5), 1045–1051. 10.1002/jor.24555 31808569 PMC7162703

[B46] Shimono K. Tung W.-e. Macolino C. Chi A. H.-T. Didizian J. H. Mundy C. (2011). Potent Inhibition of Heterotopic Ossification by Nuclear Retinoic Acid Receptor-γ Agonists. Nat. Med. 17 (4), 454–460. 10.1038/nm.2334 21460849 PMC3073031

[B55] Shukunami C. Ishizeki K. Atsumi T. Ohta Y. Suzuki F. Hiraki Y. (1997). Cellular Hypertrophy and Calcification of Embryonal Carcinoma-Derived Chondrogenic Cell Line ATDC5 *in vitro* . J. Bone Miner. Res. 12 (8), 1174–1188. 10.1359/jbmr.1997.12.8.1174 9258747

[B47] Thielen N. van der Kraan P. van Caam A. (2019). TGFβ/BMP Signaling Pathway in Cartilage Homeostasis. Cells 8 (9), 969. 10.3390/cells8090969 PMC6769927 31450621

[B48] Uchibe K. Son J. Larmour C. Pacifici M. Enomoto-Iwamoto M. Iwamoto M. (2017). Genetic and Pharmacological Inhibition of Retinoic Acid Receptor γ Function Promotes Endochondral Bone Formation. J. Orthop. Res. 35 (5), 1096–1105. 10.1002/jor.23347 27325507 PMC6900928

[B49] Urist M. R. (1965). Bone: Formation by Autoinduction. Science 150 (3698), 893–899. 10.1126/science.150.3698.893 5319761

[B50] Williams J. A. Kondo N. Okabe T. Takeshita N. Pilchak D. M. Koyama E. (2009). Retinoic Acid Receptors Are Required for Skeletal Growth, Matrix Homeostasis and Growth Plate Function in Postnatal Mouse. Dev. Biol. 328 (2), 315–327. 10.1016/j.ydbio.2009.01.031 19389355 PMC4085816

[B51] Wozney J. M. Rosen V. Celeste A. J. Mitsock L. M. Whitters M. J. Kriz R. W. (1988). Novel Regulators of Bone Formation: Molecular Clones and Activities. Science 242 (4885), 1528–1534. 10.1126/science.3201241 3201241

[B52] Yokoyama K. Ikeya M. Umeda K. Oda H. Nodomi S. Nasu A. (2015). Enhanced Chondrogenesis of Induced Pluripotent Stem Cells from Patients with Neonatal-Onset Multisystem Inflammatory Disease Occurs via the Caspase 1-independent cAMP/protein Kinase A/CREB Pathway. Arthritis Rheumatol. 67 (1), 302–314. 10.1002/art.38912 25302486

[B53] Zara J. N. Siu R. K. Zhang X. Shen J. Ngo R. Lee M. (2011). High Doses of Bone Morphogenetic Protein 2 Induce Structurally Abnormal Bone and Inflammation *In Vivo* . Tissue Engineering. Part. A. 17 (9-10), 1389–1399. 10.1089/ten.TEA.2010.0555 21247344 PMC3079169

[B54] Zhao L. Li G. Zhou G.-Q. (2009). SOX9 Directly Binds CREB as a Novel Synergism with the PKA Pathway in BMP-2-Induced Osteochondrogenic Differentiation. J. Bone Mineral Res. 24 (5), 826–836. 10.1359/jbmr.081236 19113914

