# Supplementary material for: Selective Retinoic Acid Receptor γ Antagonist 7C is a Potent Enhancer of BMP-Induced Ectopic Endochondral Bone Formation
Source: Front Cell Dev Biol. 2022 Mar 14;10:802699. doi: 10.3389/fcell.2022.802699 (PMC8963923; doi:10.3389/fcell.2022.802699)
Supplement: Supplementary file 2 [file DataSheet3.docx]

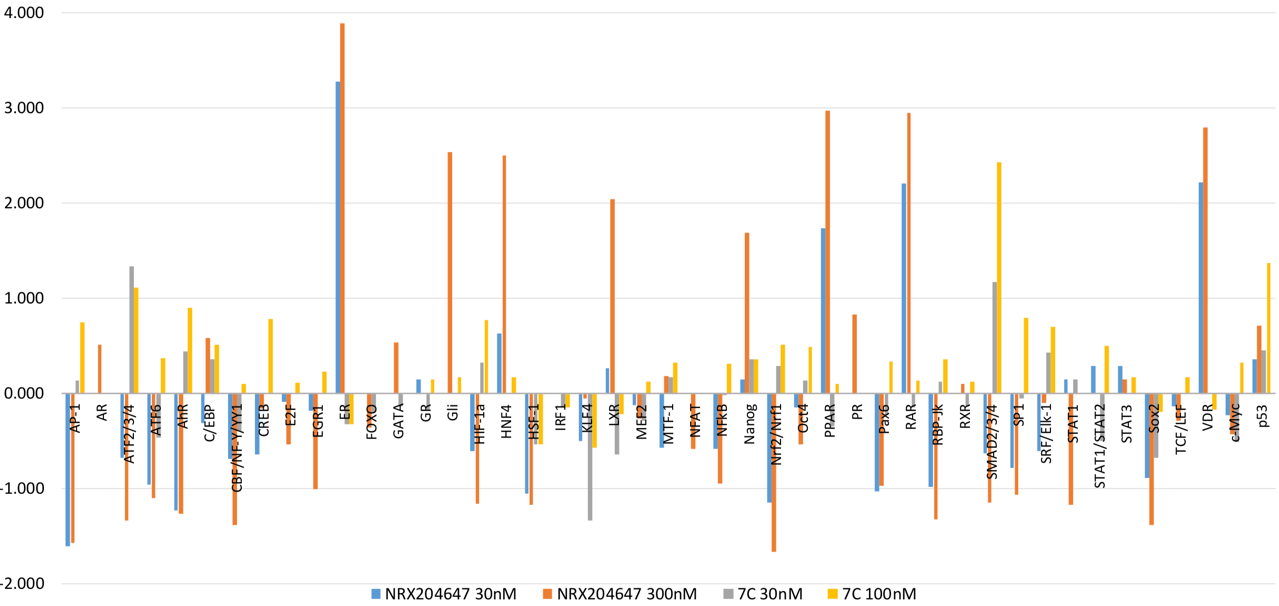

**Supplementary Figure 3.** Profiling effects of selective RARγ agonist NRX204647 and antagonist 7C. Cignal Finder 45-pathway array was used to profile the action of selective RARγ ligands. Values of drug treated groups are averages of 2 samples. Values of control are averages of samples. Please refer manufacturers handbook for detail information of pathway specific reporters and experimental procedures.
