# Supplementary material for: Selective Retinoic Acid Receptor γ Antagonist 7C is a Potent Enhancer of BMP-Induced Ectopic Endochondral Bone Formation
Source: Front Cell Dev Biol. 2022 Mar 14;10:802699. doi: 10.3389/fcell.2022.802699 (PMC8963923; doi:10.3389/fcell.2022.802699)
Supplement: Supplementary file 3 [file Table2.docx]

| Gene | Forward | Reverse |
| --- | --- | --- |
| Col2a1 | GGTGTGAAGGGTGAGAGTGG | CCTGGCTGGCCATCGTTAC |
| ACAN | AACAACTGCAGGCTGCCTAT | CCAGGGAACTCGTCCTTGTC |
| ADMTS4 | TCGTGGAGACACTGGTGGTA | GCCATAACCGTCAGCAGGTA |
| ADMTS5 | GCGCTAGACGTCCGTTACAG | CCACTGCAGCTGTGTAGAGT |
| MMP9 | CTGCGGTCCTCACCATGA | TGTCCGTGAGGTTGGAGGTT |
| Sox9 | TCCCCGCAACAGATCTCCTA | GAGCCGGAGTTCTGATGGTC |
| TNFα | TCTCAGCCTCTTCTCATTCCTGCT | AGAACTGATGAGAGGGAGGCCATT |
| IL-1β | AAAGCTCTCCACCTCAATGG | AGGCCACAGGTATTTTGTCG |
| IL-6 | AGCCAGAGTCCTTCAGAG | CCACTCCTTCTGTGACTC |
| GAPDH | GGGTGTGAACCACGAGAAAT | ACTGTGGTCATGAGCCCTTC |

**Supplementary Table 2**. Primer sequences for gene expression analysis
