# Supplementary material for: Selective Retinoic Acid Receptor γ Antagonist 7C is a Potent Enhancer of BMP-Induced Ectopic Endochondral Bone Formation
Source: Front Cell Dev Biol. 2022 Mar 14;10:802699. doi: 10.3389/fcell.2022.802699 (PMC8963923; doi:10.3389/fcell.2022.802699)
Supplement: Supplementary file 4 [file DataSheet2.docx]

**Supplementary Figure 2.** Reporter assay. (A) RARγ antagonists enhanced the BMP-2 stimulatory action of the aggrecan promoter (4xA1-p89-luc) activity. (B) Id1-luc activity. While CD2665 increased Id-luc activity in a relatively narrow dose range (100nM-1uM), 7C increased Id-luc reporter activity at all doses tested (10nM-3uM). (C) The retionid reporter assay confirmed inhibitory action of RARγ antagonists on the retinoid signaling.
