# Supplementary material for: Selective Retinoic Acid Receptor γ Antagonist 7C is a Potent Enhancer of BMP-Induced Ectopic Endochondral Bone Formation
Source: Front Cell Dev Biol. 2022 Mar 14;10:802699. doi: 10.3389/fcell.2022.802699 (PMC8963923; doi:10.3389/fcell.2022.802699)
Supplement: Supplementary file 5 [file DataSheet1.docx]

**Supplementary Figure 1.** Chemical structures of 7C (selective RARγ antagonist) and NRX204647 (selective RARγ agonist). MW, molecular weight; IC_50_, half maximal inhibitory concentration; EC50, half maximal effective concentration.
